# Assessing the Impact of Irritable Bowel Syndrome on Quality of Life in Patients at Family Medicine and Primary Health Care Clinics of the National Guard Health Affairs, Riyadh

**DOI:** 10.7759/cureus.76158

**Published:** 2024-12-21

**Authors:** Mohammed N AlDosari, Rakan M Alotaibi, Mohammad N Algahtani, Turki S Alshammari, Bader F Almziri

**Affiliations:** 1 Family and Community Medicine, King Saud Bin Abdulaziz University for Health Sciences, Riyadh, SAU

**Keywords:** ibs, ibs (irritabel bowel syndrome), irritable bowel syndrome, quality-of-life, quality of life (qol)

## Abstract

Introduction

Irritable bowel syndrome (IBS) is one of the most prevalent GI conditions, characterized by symptoms such as abdominal pain relieved by defecation, changes in bowel habits (e.g., diarrhea, constipation, or both), and bloating. These symptoms can profoundly impact the quality of life (QoL) and psychological state of patients. Despite a high prevalence in the Kingdom of Saudi Arabia, estimated at around 18.2%, there is a significant lack of studies assessing and documenting the impact of IBS on life satisfaction and the overall mental well-being of individuals within the kingdom. This study aims to bridge this gap by assessing the impact of IBS on the QoL in patients attending family medicine and primary health care clinics at National Guard Health Affairs in Riyadh, Saudi Arabia.

Methods

A total of 379 IBS patients who met the eligibility criteria participated in this cross-sectional study. The study was conducted at family medicine clinics within the National Guard Health Affairs in Riyadh. Participants were selected through a non-probability consecutive sampling technique. The Irritable Bowel Syndrome-Quality of Life Measure (IBS-QOL) questionnaire was primarily used for assessment. Both descriptive and inferential statistics were performed.

Results

In this study, males comprised 57% of the sample, and 79% were married. Forty percent held a bachelor's degree, while 30% earned between 5,000 and 9,000 riyals a month. The QoL of these individuals was profoundly affected by IBS. They frequently reported feelings of vulnerability, isolation, depression, and helplessness due to their bowel problems. The two most common concerns were monitoring dietary intake and food choices and difficulty controlling bowels in public. The IBS-QOL measure did not significantly correlate with smoking status, and no evident gender differences were found in the emotional reactions associated with IBS. Noticeable variations in the impact of IBS-QOL among age groups were observed, but no clear pattern emerged.

Conclusion

This study highlights the impact of IBS on various facets of daily living, including dietary, psychological, emotional, social, and functional aspects. Recognizing these outcomes helps in developing strategies to minimize patient suffering and enhance overall satisfaction.

## Introduction

Irritable bowel syndrome (IBS) is one of the most common gastrointestinal conditions. The syndrome affects the large intestines and is functional in nature. The condition presents with a combination of symptoms, including recurrent, nonspecific changes in bowel movements, such as diarrhea, constipation, or alternating between both states. It may also present with abdominal symptoms such as diffuse pain or bloating. Based on the symptoms affecting the patient, we can classify IBS into four different subtypes: diarrhea predominant with abdominal discomfort (IBS-D), constipation predominant with abdominal discomfort (IBS-C), alternating diarrhea or constipation with abdominal discomfort (IBS-Mixed), or undefined (IBS-U) where the symptoms vary [1,2].

The exact pathophysiology is unknown but may involve changes in gastrointestinal motility, visceral hypersensitivity, and altered gastrointestinal permeability. While the exact cause is unknown, there are known factors that contribute to triggering flares in patients affected by the condition. These factors may include psychological states like stress, depression, or anxiety, as well as consumption of certain types of food and some medications [3]. A study conducted in Northern Saudi Arabia suggested an increase in IBS prevalence in the region due to poor dietary habits. These habits may be attributed to low water intake, insufficient consumption of fiber-rich foods like fruits and vegetables, and the frequent intake of spicy and fatty foods, which are potential triggers for IBS symptoms [4]. The diagnosis of IBS is generally made clinically using the Rome IV criteria, which require that patients have experienced recurring abdominal pain associated with two or more of the following symptoms at least one day per week over the past three months: abdominal pain related to defecation, a change in stool frequency, or a change in stool form or appearance [5].

A study was conducted in Saudi Arabia to evaluate the prevalence of IBS and its subtypes among the general population. The study gathered 1,680 eligible participants from different regions in a nationwide survey. The study found the prevalence of IBS to be 18.2%, with IBS-M being the most common subtype among total patients (42.3%). As a result, the study concluded that IBS is prevalent in Saudi Arabia [6]. Furthermore, a meta-analysis published in 2019 showed the prevalence rates of anxiety symptoms and disorders in IBS patients to be 39.1% and 23% respectively. The prevalence estimates of depressive symptoms and disorders in IBS patients were 28.8% and 23.3% respectively. The study concluded that patients with IBS have a three-fold increased odds of either anxiety or depression, compared to healthy subjects [7]. Similarly, a recent study highlighted the prevalence of IBS among Saudi college students. This younger demographic faced a significant psychological burden associated with IBS, linking its prevalence to adverse effects on both their mental and physical well-being [8].

IBS not only affects patients physically but can also have a negative impact psychologically. Many factors play a role, including stress, anxiety, and depression. In addition to the psychological aspect, its effect on social life may manifest as disturbances in daily activities, social interactions, and work productivity. These factors combined can cause a significant decrease in quality of life (QoL). We should understand the impact of IBS and its associated symptoms on patients’ psychosocial health and overall QoL. Even though the prevalence of IBS is high in Saudi Arabia, there aren’t many studies documenting its impact on psychosocial health. In conclusion, improving the general well-being and QoL of those who live with this chronic condition requires a deep understanding and addressing the psychosocial effects of IBS. To provide a comprehensive approach and to effectively manage IBS and its difficulties, a complete approach that combines medical, psychological, and social support is recommended [9,10].

Due to the lack of such information in this area, we conducted this study to explore the impact of IBS on patient QoL in Ministry of National Guard Health Affairs (MNGHA), Riyadh, Saudi Arabia.

## Materials and methods

Study design

A total of 379 IBS patients who met the eligibility criteria participated in a cross-sectional study aimed at assessing the impact of IBS on individuals' QoL. The study was conducted at family medicine clinics within the National Guard Health Affairs in Riyadh.

Study population

Participants were selected through a non-probability consecutive sampling technique.

Inclusion and exclusion criteria

The study included patients diagnosed with IBS for at least six months, aged between 18 and 65 years. However, it excluded patients with other significant gastrointestinal diseases such as inflammatory bowel disease (IBD) or diverticulitis, those with severe mental health conditions like schizophrenia or bipolar disorder, and patients who had undergone recent surgical interventions. These criteria ensured the selection of a homogeneous group specifically affected by IBS, minimizing confounding variables related to other significant health conditions.

Study measures

The IBS-QOL questionnaire was primarily used for the assessment [11]. We adopted the IBS-QOL from the MAPI Research institution, which was developed by a team of researchers from the University of Washington, Seattle under the direction of Dr. Donald L. Patrick and Dr. Douglas Drossman, with sponsorship from Novartis Pharmaceuticals Corporation. Additionally, the IBS-QOL consists of 34 items, each with a five-point response scale where 1 corresponds to 'Not at all' and 5 represents 'Extreme effect'. It is interpreted across eight domains of the IBS-QOL: Dysphoria, Interference with Activity, Body Image, Health Worry, Food Avoidance, Social Reaction, Sexual, and Relationships.

Statistical analysis

An Excel spreadsheet was first used to collect the data before it was uploaded to JMP Pro for analysis. Both descriptive and inferential statistics were performed. The Chi-square test was utilized for categorical variables (e.g., gender, education), while t-tests or Mann-Whitney U tests were applied to continuous variables like the IBS-QOL scores.

Ethical clearance

A questionnaire was administered, and completion of the questionnaire by the patients was considered as consent. Participation in the study was voluntary. Participants were free to withdraw at any time, and no compensation or benefit was provided. The data collected was kept secure, with access restricted to the Principal Investigator and Co-investigators. Data entry on computers did not include any identifying information of the respondents, and data was stored in locked cabinets and password-protected computers.

## Results

Table 1 shows the sociodemographic characteristics of the studied population. It indicates that 57% were males and 79% were married. The distribution of age groups was fairly even, with 18% aged 31-35, followed by 17% aged 36-40, and 16% aged 26-30 years. Nearly 40% hold bachelor's degrees, while small numbers have primary, middle, and higher education at 4%. About 30% have an average income level of 5,000-9,000 riyals monthly, and 5% earn more than 20,000. Three-quarters of the sample reported never having smoked.

**Table 1:** Sociodemographic characteristics of the studied participants (N=379).

Variable	Count (%)
Gender	
Female	162 (42.86)
Male	216 (57.14)
Age	
18-25	48 (12.70)
26-30	64 (16.93)
31-35	71 (18.78)
36-40	67 (17.72)
41-45	43 (11.38)
46-50	36 (9.52)
51-55	26 (6.88)
56-59	8 (2.12)
60 and more	15 (3.97)
Marital status	
Single	60 (15.87)
Married	301 (79.63)
Widow	4 (1.06)
Divorced	13 (3.44)
Educational level	
Uneducated	7 (1.85)
Primary school	16 (4.23)
Middle school	16 (4.23)
High school	106 (28.04)
Diploma	67 (17.72)
Bachelor	150 (39.68)
Higher education	16 (4.23)
Income level	
Less than 5000	57 (15.08)
5000-9000	108 (28.57)
10000-20000	117 (30.95)
More than 20000	20 (5.29)
No income	76 (20.11)
Smoking	
Yes	92 (24.34)
No	286 (75.66)

Table 2 displays elements of the measure used in the study, which was the IBS-QOL. Four of the statements received the highest number of responses as "slightly" (I feel angry that I have bowel problems 27%, I feel irritable because of my bowel problems 25%, I feel unclean because of my bowel problems 28%, and I am bothered by how much time I spend on the toilet 25%). Additionally, one of the statements was most frequently answered as "moderately" (I have to watch the amount of food I eat because of my bowel problems 25%), and one statement received a high response rate as "quite a bit" (I have to watch the kind of food I eat because of my bowel problems 27%).

**Table 2:** Description of the irritable bowel syndrome-quality of life measure (IBS-QOL) statements.

Statement	Responses N (%)				
	Not at all	Slightly	Moderately	Quite a bit	Extremely
I feel helpless because of my bowel problems.	111 (29.37)	127 (33.60)	80 (21.16)	48 (12.70)	12 (3.17)
I am embarrassed by the smell caused by my bowel problems.	154 (40.74)	113 (29.89)	71 (18.78)	28 (7.41)	12 (3.17)
I feel vulnerable to other illnesses because of my bowel problems.	168 (44.44)	83 (21.96)	67 (17.72)	50 (13.23)	10 (2.65)
I feel uncomfortable when I talk about my bowel problems.	130 (34.39)	104 (27.51)	76 (20.11)	54 (14.29)	14 (3.70)
I feel depressed about my bowel problems.	158 (41.80)	87 (23.02)	70 (18.52)	50 (13.23)	13 (3.44)
I feel isolated from others because of my bowel problems.	191 (50.53)	79 (20.90)	54 (14.29)	42 (11.11)	12 (3.17)
Because of my bowel problems, sexual activity is difficult for me.	208 (55.03)	78 (20.63)	57 (15.08)	27 (7.14)	8 (2.12)
I feel angry that I have bowel problems.	87 (23.02)	103 (27.25)	78 (20.63)	69 (18.25)	41 (10.85)
I feel irritable because of my bowel problems.	83 (21.96)	98 (25.93)	69 (18.25)	89 (23.54)	39 (10.32)
I feel sluggish because of my bowel problems.	87 (23.02)	108 (28.57)	84 (22.22)	77 (20.37)	22 (5.82)
I feel unclean because of my bowel problems.	243 (64.29)	60 (15.87)	43 (11.38)	25 (6.61)	7 (1.85)
Long trips are difficult for me because of my bowel problems.	147 (38.89)	89 (23.54)	67 (17.72)	44 (11.64)	31 (8.20)
I feel frustrated that I cannot eat when I want because of my bowel problems.	110 (29.10)	92 (24.34)	75 (19.84)	64 (16.93)	37 (9.79)
It is important to be near a toilet because of my bowel problems.	167 (44.18)	83 (21.96)	68 (17.99)	36 (9.52)	24 (6.35)
I feel that no one understands my bowel problems.	126 (33.33)	103 (27.25)	66 (17.46)	45 (11.90)	38 (10.05)
	Not at all	Slightly	Moderately	Quite a bit	A great deal
I am bothered by how much time I spend on the toilet.	91 (24.07)	97 (25.66)	80 (21.16)	93 (24.60)	17 (4.50)
I feel fat because of my bowel problems.	147 (38.89)	75 (19.84)	67 (17.72)	68 (17.99)	21 (5.56)
I feel like I'm losing control of my life because of my bowel problems.	158 (41.80)	97 (25.66)	60 (15.87)	52 (13.76)	11 (2.91)
I feel my life is less enjoyable because of my bowel problems.	122 (32.28)	113 (29.89)	75 (19.84)	56 (14.81)	12 (3.17)
I have to watch the amount of food I eat because of my bowel problems.	60 (15.87)	81 (21.43)	95 (25.13)	83 (21.96)	59 (15.61)
I feel like I irritate others because of my bowel problems	146 (38.62)	88 (23.28)	68 (17.99)	51 (13.49)	25 (6.61)
I worry that my bowel problems will get worse.	100 (26.46)	98 (25.93)	71 (18.78)	79 (20.90)	30 (7.94)
I worry that people think I exaggerate my bowel problems.	149 (39.42)	69 (18.25)	71 (18.78)	55 (14.55)	34 (8.99)
I feel I get less done because of my bowel problems.	144 (38.10)	91 (24.07)	79 (20.90)	45 (11.90)	19 (5.03)
I have to avoid stressful situations because of my bowel problems	109 (28.84)	96 (25.40)	80 (21.16)	69 (18.25)	24 (6.35)
My bowel problems reduce my sexual desire.	197 (52.12)	85 (22.49)	51 (13.49)	33 (8.73)	12 (3.17)
My bowel problems limit what I can wear.	177 (46.83)	71 (18.78)	49 (12.96)	59 (15.61)	22 (5.82)
I have to avoid strenuous activity because of my bowel problems.	168 (44.44)	86 (22.75)	64 (16.93)	43 (11.38)	17 (4.50)
I have to watch the kind of food I eat because of my bowel problems.	52 (13.76)	74 (19.58)	74 (19.58)	105 (27.78)	73 (19.31)
Because of my bowel problems, I have difficulty being around people I do not know well.	180 (47.62)	75 (19.84)	65 (17.20)	42 (11.11)	16 (4.23)
My life revolves around my bowel problems.	196 (51.85)	81 (21.43)	58 (15.34)	28 (7.41)	15 (3.97)
I worry about losing control of my bowels.	138 (36.51)	109 (2.84)	59 (15.61)	50 (13.23)	22 (5.82)
I fear that I won't be able to have a bowel movement.	162 (42.86)	92 (24.34)	49 (12.96)	53 (14.02)	22 (5.82)
My bowel problems are affecting my closest relationships.	212 (56.08)	76 (20.11)	40 (10.58)	32 (8.47)	18 (4.76)

Tables 3-5 explore the association between age groups, gender, and smoking habits with IBS-QOL scores. No statistically significant differences were found across all variables.

**Table 3:** Smoking behavior in relation to some of the irritable bowel syndrome-quality of life measure statements. Statistical significance was assessed using Pearson's Chi-squared test.

Statement	Smoking N (%)		χ²	P-value a
	Yes	No		
I feel depressed about my bowel problems.				
Extremely	2 (15.38)	11 (84.62)	2.243	0.691
Quite a bit	12 (24)	38 (76)		
Moderately	15 (21.43)	55 (78.57)		
Slightly	19 (21.84)	68 (78.16)		
Not at all	44 (27.85)	114 (72.15)		
I feel angry that I have bowel problems.				
Extremely	9 (21.95)	32 (78.05)	2.6	0.627
Quite a bit	16 (23.19)	53 (76.81)		
Moderately	17 (21.79)	61 (78.21)		
Slightly	31 (30.10)	72 (69.90)		
Not at all	19 (21.84)	68 (78.16)		
I am bothered by how much time I spend on the toilet.				
A great deal	3 (17.65)	14 (82.35)	4.484	0.345
Quite a bit	20 (21.51)	73 (78.49)		
Moderately	19 (23.75)	61 (76.25)		
Slightly	31 (31.96)	66 (68.04)		
Not at all	19 (20.88)	72 (79.12)		
I have to watch the amount of food I eat because of my bowel problems.				
A great deal	13 (22.03)	46 (77.97)	7.26	0.123
Quite a bit	15 (18.07)	68 (81.93)		
Moderately	21 (22.11)	74 (77.89)		
Slightly	21 (25.93)	60 (74.07)		
Not at all	22 (36.67)	38 (63.33)		
I worry about losing control of my bowels.				
A great deal	6 (27.27)	16 (72.73)	4.317	0.365
Quite a bit	10 (20)	40 (80)		
Moderately	9 (15.25)	50 (84.75)		
Slightly	30 (27.52)	79 (72.48)		
Not at all	37 (26.81)	101 (73.19)		

**Table 4:** Gender differences in relation to some of the irritable bowel syndrome-quality of life measure statements. Statistical significance was assessed using Pearson's Chi-squared test.

Statement	Gender N (%)		χ²	P-value a
	Male	Female		
I feel depressed about my bowel problems.				
Extremely	6 (46.15)	7 (53.85)	3.087	0.543
Quite a bit	28 (56)	22 (44)		
Moderately	38 (54.29)	32 (45.71)		
Slightly	46 (52.87)	41 (47.13)		
Not at all	98 (62.03)	60 (37.97)		
I feel angry that I have bowel problems.				
Extremely	22 (53.66)	19 (46.34)	0.928	0.921
Quite a bit	41 (59.42)	28 (40.58)		
Moderately	45 (57.69)	33 (42.31)		
Slightly	56 (54.37)	47 (45.63)		
Not at all	52 (59.77)	35 (40.23)		
I am bothered by how much time I spend on the toilet.				
A great deal	10 (58.82)	7 (41.18)	0.949	0.917
Quite a bit	54 (58.06)	39 (41.94)		
Moderately	42 (52.50)	38 (47.50)		
Slightly	56 (57.73)	41 (42.27)		
Not at all	54 (59.34)	37 (40.66)		
I have to watch the amount of food I eat because of my bowel problems.				
A great deal	32 (54.24)	27 (45.76)	3.828	0.43
Quite a bit	47 (56.63)	36 (43.37)		
Moderately	51 (53.68)	44 (46.32)		
Slightly	45 (55.56)	36 (44.44)		
Not at all	41 (68.33)	19 (31.67)		
I worry about losing control of my bowels.				
A great deal	13 (59.09)	9 (40.91)	4.054	0.399
Quite a bit	25 (50)	25 (50)		
Moderately	30 (50.85)	29 (49.15)		
Slightly	61 (55.96)	48 (44.04)		
Not at all	87 (63.04)	51 (36.96)		

**Table 5:** Age group differences in relation to some of the irritable bowel syndrome-quality of life measure statements. Statistical significance was assessed using the Fisher-Freeman-Halton exact test due to small expected cell frequencies.

Statement	Age groups N (%)					Test value	P-value a
	18-30	31-40	41-50	51-60	<60		
I feel depressed about my bowel problems.							
Extremely	7 (53.85)	3 (23.08)	2 (15.38)	1 (7.69)	0	10.105	0.847
Quite a bit	12 (24)	17 (34)	12 (24)	6 (12)	3 (6)		
Moderately	16 (22.86)	27 (38.57)	18 (25.71)	7 (10)	2 (2.86)		
Slightly	26 (29.89)	36 (41.38)	16 (18.39)	5 (5.75)	4 (4.60)		
Not at all	51 (32.28)	55 (34.81)	31 (19.62)	15 (9.49)	6 (3.80)		
I feel angry that I have bowel problems.							
Extremely	13 (31.71)	18 (43.90)	8 (19.51)	2 (4.88)	0	12.958	0.665
Quite a bit	18 (26.09)	29 (42.03)	15 (21.74)	3 (4.35)	4 (5.80)		
Moderately	22 (28.21)	26 (33.33)	16 (20.51)	12 (15.38)	2 (2.56)		
Slightly	32 (31.07)	39 (37.86)	20 (19.42)	9 (8.74)	3 (2.91)		
Not at all	27 (31.03)	26 (29.89)	20 (22.99)	8 (9.20)	6 (6.90)		
I am bothered by how much time I spend on the toilet.							
A great deal	4 (23.53)	6 (35.29)	2 (11.76)	5 (29.41)	0	18.463	0.26
Quite a bit	28 (30.11)	29 (31.18)	23 (24.73)	9 (9.68)	4 (4.30)		
Moderately	23 (28.75)	33 (41.25)	14 (17.50)	9 (11.25)	1 (1.25)		
Slightly	27 (27.84)	43 (44.33)	17 (17.53)	5 (5.15)	5 (5.15)		
Not at all	30 (32.97)	27 (29.67)	23 (25.27)	6 (6.59)	5 (5.49)		
I have to watch the amount of food I eat because of my bowel problems.							
A great deal	18 (30.51)	23 (38.98)	13 (22.03)	5 (8.47)	0	9.455	0.899
Quite a bit	26 (31.33)	28 (33.73)	15 (18.07)	10 (12.05)	4 (4.82)		
Moderately	28 (29.47)	39 (41.05)	18 (18.95)	6 (6.32)	4 (4.21)		
Slightly	25 (30.86)	27 (33.33)	16 (19.75)	9 (11.11)	4 (4.94)		
Not at all	15 (25)	21 (35)	17 (28.33)	4 (6.67)	3 (5)		
I worry about losing control of my bowels.							
A great deal	8 (36.36)	8 (36.36)	3 (13.64)	3 (133.64)	0	13.81	0.585
Quite a bit	15 (30)	18 (36)	12 (24)	4 (8)	1 (2)		
Moderately	19 (32.20)	18 (30.51)	16 (27.12)	3 (5.08)	3 (5.08)		
Slightly	23 (21.10)	49 (44.95)	20 (18.35)	13 (11.93)	4 (3.67)		
Not at all	47 (34.06)	45 (32.61)	28 (20.29)	11 (7.97)	7 (5.07)		

## Discussion

IBS is one of the most common GI conditions. Despite numerous studies on the worldwide prevalence and etiology of IBS, there is insufficient data on the psychosocial impact among the general population of Saudi Arabia. The purpose of this study is to evaluate the psychosocial impact on patients complaining of IBS who attended the National Guard Health Affairs in Riyadh.

In our sample, we noted a significant degree of impairment and avoidance of daily activities related to IBS. A major impact on their QoL was primarily observed in terms of food habits. Food has been reported as one of the most common triggers of IBS; approximately 47.09% reported avoidance or monitoring of certain types of food, while 37.57% reported a change in the amount of food they consumed. It was shown that food habits are directly linked to how IBS patients experience their symptoms. IBS patients with regular dietary habits experience a lesser degree of symptom severity. In contrast, those with irregular dietary habits tend to report much greater severity of symptoms, which correlates with a higher impact on QoL. A study in Northern Saudi Arabia revealed that inadequate water consumption and a lack of sufficient fiber, particularly from fruits and vegetables, may contribute to the rising prevalence of IBS in Saudi Arabia [4]. This is consistent with the National Institute for Health and Care Excellence (NICE) guidelines that recommend regular small meals, avoiding gas-producing vegetables, limiting fiber intake, and reducing coffee and tea consumption to alleviate IBS symptoms [12]. Our study did not evaluate how adherence to certain diets could improve QoL. Future studies should focus on assessing the effects of implementing dietary interventions within the Saudi population to better understand the impact on outcomes.

Exploring the psychological aspect, our patients express a profound toll from their bowel problems. A majority reported various concerns reflecting anxiety and worry about many aspects of their condition. Generally, concerns regarding disease progression and symptom management have been highlighted as primary concerns by our patients. Many fear the inability to have a bowel movement, while others fear losing control of their bowels. As IBS includes a wide range of symptoms, these concerns were reported at similar rates in our study despite being opposites. Many more fear their bowel symptoms will worsen. Moreover, a smaller subset have expressed feelings of helplessness regarding their symptoms. Several participants reported feelings of depression and isolation because of their bowel problems. These findings reveal the multifaceted psychological burden that individuals with IBS endure, influencing their daily lives and overall mental health. This aligns with the theory that patients with IBS are associated with higher degrees of anxiety and depression. A systematic review that included 73 papers found that patients with IBS have a three-fold increased odds of either anxiety or depression compared to healthy subjects [7].

In terms of emotional response, patients reported a major emotional impact due to their bowel problems. For example, a subset of patients reported frustrations regarding the inability to eat what they wanted. Others reported experiencing feelings of irritability and anger about their condition. Research suggests that anger plays a role in affecting the QoL of IBS patients through the emotional scheme model. Similar to these theories, our results reach a consensus in supporting the positive relationship between IBS and emotional regulation [13]. Depending on how individuals process their anger and emotions, whether it is adaptive (helpful) or non-adaptive (unhelpful), it can exacerbate the severity of symptoms, making their QoL worse. Other studies also highlighted a similar outcome in terms of emotional response, with patients having decreased emotional resilience linked to a higher degree of IBS exacerbation and increase in symptom severity [14]. These elements highlight the profound emotional toll IBS can have on patients, impacting their overall QoL and mental well-being.

When assessing the social impact of IBS, our patients reported a significant social burden caused by their bowel problems. The majority indicated they have to avoid stressful situations, aligning with clinical evidence that stress induces alterations in neuro-endocrine-immune pathways disturbing the gut-brain axis, causing a flare-up in IBS and eventually leading to a decrease in QoL [15]. Additionally, some individuals feel overweight due to their condition, likely because bloating is one of the cornerstone symptoms of IBS. This corresponds with findings that abdominal bloating is associated with increased symptoms and pain severity, thus impacting QoL and general well-being [16]. Moreover, patients also reported difficulty being around people they do not know well, and some noted that their bowel problems are affecting their closest relationships. Recognizing the potential interpersonal impact of social and close relationships is important in relation to QoL, as IBS is known to affect interpersonal relationships. This might be due to social isolation, interpersonal conflicts, disease stigma, and the intrusiveness that comes with being in a relationship, coinciding with the embarrassing and socially taboo nature of the symptoms [17].

On evaluating the functional QoL, patients reported that their condition limits their choices in clothing, mainly by avoiding tight clothes, as it becomes restricting when patients experience bloating and gas build-up in the intestine. They also reported experiencing feelings of embarrassment and uncleanliness due to the smell caused by their bowel problems. A study set in Al Qunfudah, Saudi Arabia, concluded that IBS patients experience significant social isolation and avoidance behavior that could potentially trigger symptoms [18]. Additionally, they reported that bowel problems reduced their sexual desire and sexual activities. The decline in sexual function was attributed to symptoms of bloating, flatulence, soiling, and abdominal pain. These results are consistent with a systematic review published by Jeanette Sørensen that showed significant sexual dysfunction in patients suffering from IBS when compared to healthy controls [19]. Another study conducted in al-Jouf highlighted a higher prevalence of sexual dysfunction across the female population suffering from IBS [20].

On the other hand, some patients found that long trips are difficult, and a major factor that affected their QoL is the need to know where public bathrooms are when going out of their homes [21]. A qualitative study by Lauren White exploring the unpredictability of IBS symptoms revealed that for some patients, knowing the locations of public bathrooms is a necessity for them. This includes planning journeys, mapping out toilets, and depending on restroom facilities. As a result, they became increasingly sluggish and started to do fewer activities because of these limitations.

Our study has several limitations that must be taken into consideration. One major limitation was the use of a cross-sectional design and the many biases that come with such a design, such as social desirability, self-reporting, and recall bias. To address and mitigate these biases, a longitudinal design could be implemented in future studies, focusing on collecting data over time and continuous follow-up, which would help minimize recall and social desirability biases. Moreover, a limitation that hindered our study was the refusal of participation by some individuals who found the questions intrusive or invading their privacy. Future studies could address this matter by providing stronger assurances regarding confidentiality to minimize any fears or concerns that patients may have. Another limitation was the exclusion of patients with other significant GI diseases, such as inflammatory bowel disease, diverticulitis, and celiac disease. Subsequent research could benefit from including these populations to explore any potential overlap in terms of QoL and to allow for better generalization of findings.

## Conclusions

IBS is a common disorder among the Saudi population. In our study, patients at the National Guard Health Affairs Hospital in Riyadh showed significant impairment in the psychosocial aspects of life and avoidance of daily activities. Patients reported considerable restrictions in food choices and dietary options, as well as a notable impact on their emotional well-being, leading to depression and anxiety. This significant impact led to challenges in social relationships due to the burden of the disease on QoL. Addressing these findings is crucial for developing targeted interventions to improve the overall QoL for IBS patients in Saudi Arabia.
